# The differential impact of face distractors on visual working memory across encoding and delay stages

**DOI:** 10.3758/s13414-024-02895-6

**Published:** 2024-05-31

**Authors:** Chaoxiong Ye, Qianru Xu, Zhihu Pan, Qi-Yang Nie, Qiang Liu

**Affiliations:** 1 Institute of Brain and Psychological Sciences, Sichuan Normal University, Chengdu, 610066 China; 2 Department of Psychology, University of Jyvaskyla, Jyväskylä, 40014 Finland; 3 Center for Machine Vision and Signal Analysis, University of Oulu, Oulu, 90014 Finland; 4 Centre for Cognitive and Brain Sciences, University of Macau, Macau, 999078 China; 5 School of Education, Anyang Normal University, Anyang, 455000 China

**Keywords:** Visual short-term memory, Facial distractor, Encoding stage, Delay stage, Distraction effect

## Abstract

External distractions often occur when information must be retained in visual working memory (VWM)—a crucial element in cognitive processing and everyday activities. However, the distraction effects can differ if they occur during the encoding rather than the delay stages. Previous research on these effects used simple stimuli (e.g., color and orientation) rather than considering distractions caused by real-world stimuli on VWM. In the present study, participants performed a facial VWM task under different distraction conditions across the encoding and delay stages to elucidate the mechanisms of distraction resistance in the context of complex real-world stimuli. VWM performance was significantly impaired by delay-stage but not encoding-stage distractors (Experiment 1). In addition, the delay distraction effect arose primarily due to the absence of distractor process at the encoding stage rather than the presence of a distractor during the delay stage (Experiment 2). Finally, the impairment in the delay-distraction condition was not due to the abrupt appearance of distractors (Experiment 3). Taken together, these findings indicate that the processing mechanisms previously established for resisting distractions in VWM using simple stimuli can be extended to more complex real-world stimuli, such as faces.

**Supplementary information:**

The online version contains supplementary material available at 10.3758/s13414-024-02895-6.

## Introduction

Visual working memory (VWM) is a key cognitive system dedicated to the active retention and manipulation of a limited amount of visual information over short periods (Luck & Vogel, 1997, 2013). This system is pivotal for integrating perceptual information, and it contributes to a dynamic and coherent visual experience. A fundamental aspect of VWM is its ability to resist perceptual distractions by enabling a focus on relevant information and by filtering out extraneous stimuli. This ability is crucial in a variety of cognitive functions and everyday activities, including learning, reasoning, driving safety, and social interactions. Earlier research on VWM primarily focused on the mechanisms involved in processing the information retained in VWM (Cowan, 2001; Luck & Vogel, 1997; Olson & Jiang, 2002; Vogel et al., 2001). However, recent studies are increasingly examining the impact of distractive information on the actual functioning of VWM (Duan et al., 2023; Feldmann-Wustefeld & Vogel, 2019; Hakim et al., 2020).

Previous VWM research has revealed that distractions can arise at various stages, either during the encoding stage, when perceptual distractors are presented alongside targets, or exclusively during the delay stages (Feldmann-Wustefeld & Vogel, 2019; McNab & Dolan, 2014). In the encoding stage, a critical factor for countering distractions is the ability to selectively encode relevant information (Fukuda & Vogel, 2009, 2011; Liesefeld et al., 2020; McNab & Klingberg, 2008), and event-related potential (ERP) techniques have been instrumental in showing how participants resist distractions during encoding in VWM tasks (Feldmann-Wustefeld & Vogel, 2019; Vogel et al., 2005). Specifically, one ERP component, known as contralateral delay activity (CDA), reflects the visual information load in VWM and has been used to investigate the relationship between an individual’s ability to resist distractions at the encoding stage and the individual’s overall VWM capacity. Vogel et al. (2005) found that participants with low VWM capacities were more likely to store simple perceptual distractors (e.g., color and orientation) in VWM, while those with high VWM capacities showed effective resistance to these same distractors. This suggests that a correlation exists between an individual’s VWM capacity and the ability to resist distractors at the encoding stage. Similarly, research that has investigated resistance to more naturalistic distractors, such as facial stimuli, has revealed that individuals with lower VWM capacities struggle more to resist complex real-world distractors (Ye et al., 2018).

Some studies have gone beyond the examination of perceptual distractors presented during the encoding stage to include the effects of these distractors when they appear during the delay stage of VWM tasks (Hakim et al., 2020; McNab & Dolan, 2014). For example, Hakim et al. (2020) engaged their participants in a change detection task that required to remember six simple stimuli. In the distraction condition, the perceptual distractors occurred during the delay stage, after the memory array disappeared and before the probe array appeared. They found a decline in task performance under the distraction condition compared with the no-distraction condition, indicating that distractions were also impactful when presented exclusively during the delay stage. Further studies have suggested that the process of resisting distractors and preventing unwanted information from being encoded during the delay stage may be related to an individual’s internal attention control processes on VWM (R. Liu et al., 2023; Makovski & Jiang, 2007; Pinto et al., 2013; Rerko et al., 2014; van Moorselaar et al., 2014, 2015).

Not surprisingly, this resistance to distractors during encoding versus delay stages could uniquely contribute to VWM capacity (McNab & Dolan, 2014). For instance, Duan et al. (2023) recently conducted a systematic investigation into the resilience of individual VWM against perceptual distractors at both the encoding and delay stages. Their series of experiments used a delayed estimation task in which participants were asked to remember simple stimuli (e.g., the orientations of teardrops) under different distraction conditions and demonstrated different effects of distractions presented during the encoding versus the delay stage. Unexpectedly, however, VWM performance was significantly impaired only by the perceptual distractors presented during the delay stage. Distractions that occurred solely during the encoding stage, alongside memory stimuli, did not detrimentally affect VWM performance. Follow-up experiments that included a full-distraction condition in which perceptual distractors persisted throughout both the encoding and delay stages revealed that processing distractors presented during encoding could mitigate their distracting effect during the delay stage. Integrating these findings, Duan et al. (2023) proposed a two-stage Bayesian model, positing that task relevance and visual uncertainty are key factors that govern cognitive resource allocation in VWM tasks. This model effectively synthesizes the observed behaviors across the experiments and offers a nuanced understanding of how VWM resists distractions under different conditions. However, according to our knowledge, these distinct distraction effects during the encoding and delay stages have only been tested with simple stimuli (e.g., orientations of teardrops). Consequently, the inclusion of complex real-world stimuli could provide new insights into how distinct distraction effects might apply in more naturalistic VWM settings.

When considering the diverse visual inputs of the real world, human beings show a unique proficiency for processing faces, as these attract attention more effectively than most other meaningful objects (Ro et al., 2001; Vuilleumier, 2000; Young & Burton, 2018). This specialized processing of faces commences at the initial perceptual stages; consequently, face stimuli are frequently used as distractors during the encoding stage in VWM studies, especially those investigating the distraction effects of complex real-world stimuli (Stout et al., 2013, 2015; Ye et al., 2023; Ye et al., 2018). However, as is often experienced in everyday scenarios (e.g., trying to remember new acquaintances at a social gathering), the intrusion of other unfamiliar faces can disrupt the memory of the first ones. Thus, in real life, face-related distractions are likely to occur both during the encoding of targets and during the subsequent delay stage. A recent study by Mallett et al. (2020), which explored the impact of presenting face distractors during the delay stage on VWM, reported that these distractions bias the VWM for faces. However, to the best of our knowledge, although previous studies have used faces as perceptual distractors during either the encoding or delay stages, no systematic investigation has yet examined the potential differences in the impacts of face distractors presented at the two stages.

Given the special social importance of facial stimuli and their evolutionary necessity for human survival, a natural question to ask is whether the mechanisms established for the processing of simple stimuli can be generalized to complex real-world stimuli, such as faces. For instance, marked differences are evident between simple and complex real-world stimuli when using relative positional relationships between memory items in VWM tasks to enhance memory for stimuli (Gao et al., 2016; X. Liu et al., 2022). Therefore, a thorough examination of the distraction effects of face stimuli, whether introduced during the encoding or delay stages, is needed to deepen our understanding of the influence of these stimuli on VWM tasks.

Our aim in the current study was to use a change detection task with face stimuli (i.e., a facial VWM task) to directly assess the differential impact on VWM when face distractors are presented during the encoding versus the delay stages. Two potential hypotheses were viewed as possible for the experimental results. The first hypothesis was that the influence of face distractors across different stages mirrors that of simple distractors, leading to findings akin to those published by Duan et al. (2023). In this case, when face distractors are presented during the encoding stage, they will not harm VWM performance. Only when face distractors appear during the delay stage will a decrease in VWM performance be apparent. The second hypothesis was that complex real-world stimuli, such as face distractors, are more likely than simple stimuli to disrupt VWM; therefore, the presence of face distractors, whether during the encoding or delay stage, will always harm VWM performance. Testing these hypotheses would therefore provide a deeper comprehension of how face distractors influence individual VWM and would determine whether the processing mechanisms identified for simple stimulus distractions are extendable to complex real-world distractions.

The overall goal of this study was to enrich the broader consensus on the interplay between distraction resistance and VWM. In doing so, the findings could prove vital in furthering socio-affective cognition research and thereby offer significant insights into the psychological underpinnings of a range of emotional and cognitive disorders.

## Experiment 1

To test whether the distraction effects differ when face distractors appear during the encoding stage versus the delay stage, the participants were asked to remember two target faces each time, while ignoring the face distractors regardless of when they appeared. We manipulated the conditions under which the face distractors appeared to include three different distraction conditions: a no-distraction condition, an encoding-distraction condition, and a delay-distraction condition. In the no-distraction condition, no face distractors appeared. In the encoding-distraction condition, two additional face distractors appeared alongside the target faces during the encoding stage. In the delay-distraction condition, the two additional face distractors appeared during the delay stage. This setting enabled a comparison of VWM performance under different distraction conditions (during either the encoding stage or the delay stage) against a no-distraction condition. If the presence of face distractors during the encoding or delay stages induced a significant distraction effect, the VWM performance in that distraction condition was expected to be significantly worse than in the no-distraction condition. Notably, in our study, the methodology we used to distinguish between target and distractor stimuli diverged from that of Duan et al. (2023), who used red or green teardrops as distractor stimuli (with one color designating the target and the other the distractor). Given that a teardrop is a dual-feature stimulus in which color and orientation features are bound at the same location, the participants inevitably encoded both the target shape (orientation information) and color when discriminating between the target and the distractor. However, in our study, we utilized rectangular borders framing faces to inform participants which faces were the memory targets and which were distractor stimuli. This setup allowed participants to initially encode and select targets or distractors based solely on the color of the rectangular borders without needing to automatically encode the distractor face identities (akin to the content of VWM targets). Therefore, if participants had the ability to suppress distraction effects, our paradigm could make it easier for the participants to suppress face information within the distractor stimuli. Moreover, the experimental paradigm employed by Duan et al. (2023) was a delayed estimation task, which, although potentially more sensitive than the commonly utilized VWM task of change detection, is predominantly suited for assessing simple features capable of continuous variation (e.g., color or orientation). The delayed estimation task also necessitates precise memory of the target stimuli for successful task completion, thereby potentially rendering participants more susceptible to distraction by novel stimuli during the delay stage. By contrast, our study utilized a change detection task as the participants’ VWM task. The change detection task enables the maintenance of low-precision representations of target faces that are sufficient for task performance, thereby potentially mitigating the impact of novel face distractors during the delay stage should such mitigation be feasible.

### Methods

#### Participants

Adequate statistical power for the *t*-test comparison was ensured by conducting an a priori power analysis. This analysis, performed using G*Power 3.1.9.2 (Faul et al., 2007), was based on the predicted effect size derived from the study by Duan et al. (2023). Anticipating a large effect size (Cohen’s *d* = 0.80) for our experimental design, and setting a statistical power of 80% alongside an alpha level of 0.05, the analysis recommended a total sample size of 15 participants.

Our study was conducted following the tenets of the Declaration of Helsinki and was approved by the ethics committee of Sichuan Normal University. Twenty-six college student volunteers (nine males and 17 females; mean age = 19.61 ± 1.444 years, age range: 18–24 years) participated in this study in return for compensation. This sample size aligned closely with the sample size (*N* = 24) used in the study by Duan et al. (2023). All participants reported having normal or corrected-to-normal vision, normal color vision, and no history of neurological problems. Each participant provided written informed consent before participating in the study.

### Materials

The stimuli used in the facial VWM task were 18 images of neutral male faces selected from the Chinese Facial Affective Picture System (CFAPS; Gong et al., 2011). The CFAPS has been widely used to investigate human face processing in China (Guo et al., 2013; Y. Liu et al., 2014; Luo et al., 2010; Tian et al., 2018; Ye et al., 2018; Zheng et al., 2015). The images in the CFAPS are all similar in size, background, spatial frequency, contrast grade, brightness, and other physical properties. Each selected image had a high agreement rate in terms of emotion categorization (more than 70% agreement rate for each neutral expression image). Faces were presented on a gray background and were framed with rectangular borders (2.6° wide × 3° tall). Both the memory and test arrays contained facial images placed in fixed locations surrounding a fixation cross. All faces were displayed in a memory array in an 11° × 8.2° region surrounding the fixed cross. The distance between any two faces was at least 4.6° (center-to-center). The experimental task was programmed using the E-Prime software (E-Prime 2.0, Psychology Software Tools, Inc.). Participants were seated in a dark, soundproof room at a distance of 70 cm from a 17-inch screen.

### Procedure

**Fig. 1:**
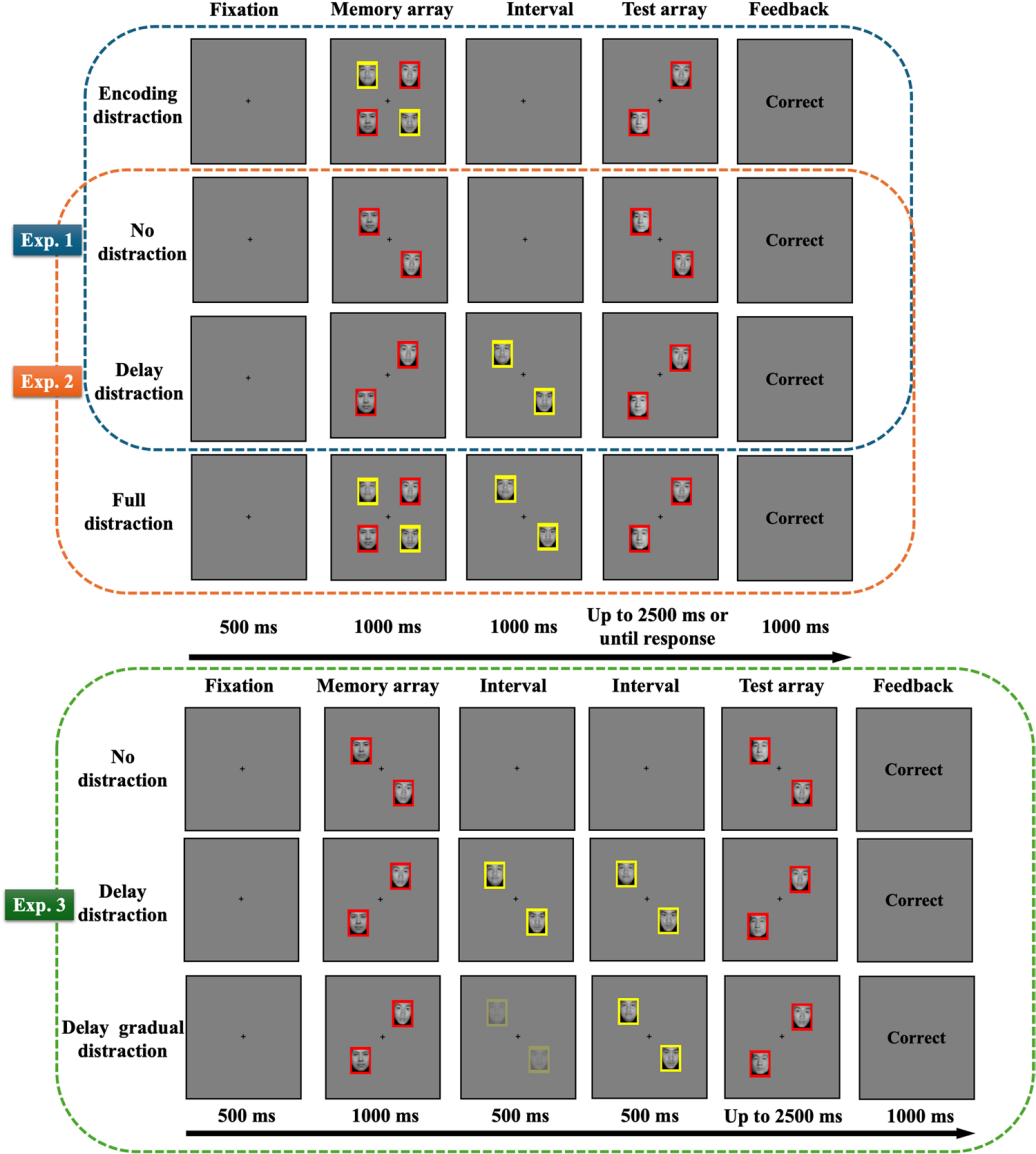
Trial structure of the facial VWM task of Experiment 1 (no-distraction condition, encoding-distraction condition, and delay-distraction condition), Experiment 2 (no-distraction condition, full-distraction condition, and delay-distraction condition), and Experiment 3 (no-distraction condition, delay-distraction condition, and delay-gradual-distraction condition). Red frames indicate targets to be memorized, and yellow frames indicate distractors. Here, only trials with identity changes are demonstrated. (Color figure online)

The trial structure of Experiment 1 is shown in Fig. 1. Participants were required to conduct a facial VWM task. Each trial began with a fixation cross in the center of the screen. After an interval (500 ms), a memory array of faces was displayed (1,000 ms). Following the memory array, an interval (1,000 ms) preceded the onset of the test array. The test array contained two facial stimuli. The test array in 50% of the trials had one face that differed from the target faces in the memory array; the test array faces were identical to the target faces in the remaining trials. The participant’s task was to indicate whether the faces in the test array were identical to the target faces in the memory array or whether a face had changed in the corresponding location between the memory and test arrays. The instructions emphasized response accuracy rather than response speed. The test array was exposed for up to 2,500 ms or until the participant responded. Following the response, feedback (1,000 ms) about the correctness of the participant’s response would appear. After the feedback disappeared, a variable interval (500–1,000 ms) elapsed before the beginning of the next trial. The experiment included three different conditions: a no-distraction condition, an encoding-distraction condition, and a delay-distraction condition. (1) In the no-distraction condition, only two target faces were presented in the memory array, followed by a blank screen during the interval, and no distractor faces were presented. (2) In the encoding-distraction condition, when the memory array appeared, two distractor faces appeared on the screen in addition to the two target faces. When the memory array disappeared, both the target and distractor faces disappeared at the same time, followed by a blank screen during the interval. (3) In the delay-distraction condition, only two target faces appeared in the memory array. After the memory array disappeared, two distractor faces appeared during the interval. The distractor faces then disappeared when the test array appeared. The target faces and distractor faces were surrounded by red or yellow frames (target or distractor frames, counterbalanced across participants). Participants were asked to remember only the identities of faces surrounded by the target frames and to ignore the faces surrounded by the distractor frames. The identities of distractors were always different from those of the target faces.

Participants completed 96 trials for each condition (no-distraction, encoding-distraction, and delay-distraction) for a total of 288 trials. Trials of each condition were fully randomized in the experiment. Instructions at the beginning of the experiment informed the participants about the task. At least 24 practice trials were performed prior to recording the test performance. The entire task lasted approximately 45 min.

### Data analysis

A repeated-measures analysis of variance (ANOVA), with conditions (no-distraction vs. encoding-distraction vs. delay-distraction) as a within-subject factor, was conducted for the accuracy (ACC). Partial eta squared (η_p_^2^) measures were used for effect size estimations for the ANOVAs. The significant main effect found in ANOVAs was followed up by applying paired *t* tests to compare the results between different conditions. We also applied the Holm–Bonferroni method to correct the original* p* values to *p*_corr_ derived from post hoc *t* tests. Cohen’s *d* was used as an estimator of the effect size of significant results in the *t* tests. Bayes factor analyses were used to show whether the ANOVA and *t*-test results supported the alternative hypothesis or the null hypothesis (Rouder et al., 2009). The Bayes factor (BF_10_) provides an odds ratio for alternative/null hypotheses (values <1 favor a null hypothesis and values >1 favor an alternative hypothesis); for example, a BF_10_ of 0.25 would indicate that the null hypothesis is four times more likely than the alternative hypothesis.

### Results

**Fig. 2:**
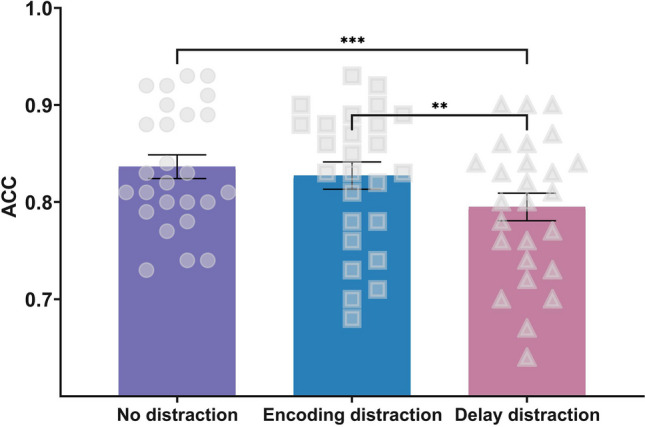
Accuracy under each condition (no-distraction condition, encoding-distraction condition, and delay-distraction condition) in Experiment 1. Mean values, with error bars showing the standard error of the mean. **p < .01, ***p < .001

The mean accuracy in each condition (no-distraction condition vs. encoding-distraction condition vs. delay-distraction condition) is shown in Fig. 2. The ANOVA for the accuracy of the responses showed a significant main effect of condition, *F*(2, 50) = 9.735, *p* < 0.001, η_p_^2^ = 0.280, BF_10_ = 95.167. The accuracy was significantly lower in the delay-distraction condition (*M* = 0.795, *SD* = 0.072) than in the encoding-distraction condition (*M* = 0.827, *SD* = 0.072), *t*(25) = 3.270, *p*_corr_ = 0.006, Cohen’s *d* = 0.641, BF_10_ = 12.717, and in the no-distraction condition (*M* = 0.837, *SD* = 0.062), *t*(25) = 4.045, *p*_corr_ < 0.001, Cohen’s *d* = 0.793, BF_10_ = 70.84. No significant difference was detected in the accuracy between the encoding distraction and no-distraction conditions, *t*(25) = 0.972, *p*_corr_ = 0.340, Cohen’s *d* = 0.191, BF_10_ = 0.318.

### Discussion

The results of Experiment 1 reveal that the participants’ VWM performance did not decrease under the encoding-distraction condition. However, a significant impairment in VWM performance was observed under the delay-distraction condition compared to the no-distraction condition. This pattern of results is consistent with that obtained by Duan et al. (2023) using simple distracting stimuli. These findings suggest that face distractors presented during the delay stage significantly disrupt VWM performance. Intriguingly, this effect of distraction was absent when the face distractors were presented during the encoding stage, indicating a distinct influence of face distraction on VWM performance at different stages. However, these results do not clarify whether the impairment in VWM performance in the delay-distraction condition is truly due to the presence of face distractors during the delay or whether it arises due to the absence of processing distractors at encoding.

Furthermore, a potential explanation for the findings of Experiment 1 might lie in the differential consolidation time of face distractors presented at different distraction conditions. Previous research has shown that the storage of complex stimuli in VWM is a sequential consolidation process (Becker et al., 2013; Hao et al., 2018; T. Liu & Becker, 2013) that prevents parallel consolidation of both target and distractor faces. When face distractors are presented during the encoding phase, the participants might unconsciously store both target and distractor faces. However, due to the sequential nature of consolidation, participants must first complete the consolidation of target faces, thereby reducing the time available for consolidating distractors and subsequently diminishing the impact of distractors on VWM during the encoding stage. By contrast, when distractors are presented only during the delay stage, participants have the opportunity to immediately begin consolidating these distractors into VWM, providing a longer consolidation period than is possible in the encoding-distraction condition. This maximizes the potential impact of distractors during the delay stage and leads to a more significantly detrimental effect on VWM performance compared with the encoding-distraction condition. Hence, the distraction in both encoding- and delay-distraction conditions is not solely attributable to the stage at which distractors are presented. It may also be influenced by the varying consolidation times of the distractors under different distraction conditions. For further clarification, we examined this possibility in Experiment 2.

## Experiment 2

In Experiment 2, we modified the encoding-distraction condition in Experiment 1 by introducing a so-called full-distraction condition. In this full-distraction condition, the face distractors were present throughout both the encoding and delay stages, effectively doubling the exposure time of the face distractors compared with the distraction conditions in Experiment 1. Assuming that the participants did indeed try to consolidate the face distractors into VWM during the encoding stage, this full-distraction setup ensured that the participants had sufficient time for the VWM consolidation of the face distractors.

Two hypotheses can be proposed. First, if VWM performance is unaffected in the full-distraction condition compared to the no-distraction condition, this would imply that the distraction effect is not related to the duration of exposure to the face distractors; rather, it is primarily due to the lack of encoding of face distractors at the encoding stage. Second, if the full-distraction condition also leads to a significant decrease in VWM performance, this would suggest that the extent of the distraction effect is related to the time allocated for VWM consolidation of the face distractors. This would also indicate that the face distractors shown during the delay stage are unconsciously consolidated and that the distraction effect observed in Experiment 1 can be attributed to the presence of face distractors during this delay stage.

### Methods

#### Participants

A new sample of 26 college students (four males and 22 females; mean age = 20.19 ± 1.918 years, age range: 18–24 years) participated in Experiment 2 in return for compensation. All participants reported having normal or corrected-to-normal vision, normal color vision, and no history of neurological problems. Each participant provided written informed consent before participating in the study.

### Procedure

The trial structure of Experiment 2 is shown in Fig. 1. The design and procedure of Experiment 2 were identical to those of Experiment 1, except for the following change: The encoding-distraction condition was replaced by the full-distraction condition. In the full-distraction condition, when the memory array appeared, two distractor faces appeared on the screen, in addition to the two target faces. When the memory array disappeared, only the target faces disappeared. Thus, the distractor faces appeared from the onset of the memory array until the test array appeared (i.e., the distractor faces were presented for 2,000 ms).

### Results

**Fig. 3:**
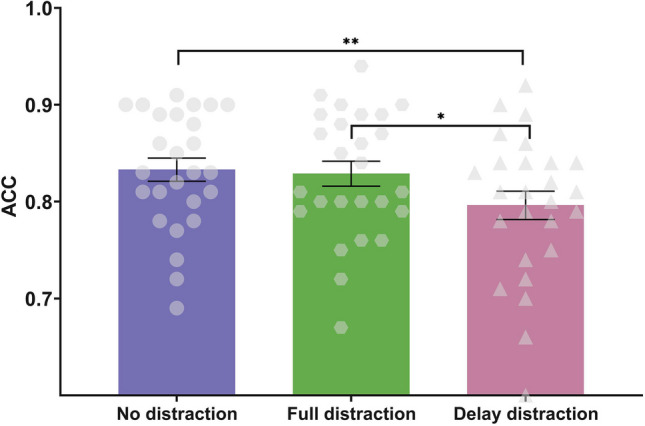
Accuracy under each condition (no-distraction condition, full-distraction condition, and delay-distraction condition) in Experiment 2. Mean values, with error bars showing the standard error of the mean. *p < .05, **p < .01

The mean accuracy in each condition (no-distraction condition vs. full-distraction condition vs. delay-distraction condition) is shown in Fig. 3. The ANOVA for the accuracy of the responses showed a significant main effect of condition, *F*(2,50) = 6.272, *p* = .004, η_p_^2^ = 0.201, BF_10_ = 10.168. The accuracy was significantly lower in the delay-distraction condition (*M* = 0.796, *SD* = 0.075) than in the full-distraction condition (*M* = 0.829, *SD* = 0.066), *t*(25) = 2.648, *p*_corr_ = 0.028, Cohen’s *d* = 0.519, BF_10_ = 3.596, and in the no-distraction condition (*M* = 0.833, *SD* = 0.061), *t*(25) = 3.429, *p*_corr_ = 0.006, Cohen’s *d* = 0.673, BF_10_ = 17.922. No significant difference was detected in the accuracy between the full-distraction and no-distraction conditions, *t*(25)= 0.383, *p*_corr_ = 0.705, Cohen’s *d* = 0.075, BF_10_ = 0.222.

### Discussion

The accuracy results indicate that the participants’ VWM performance was not significantly impaired under the full-distraction condition. However, a significant impairment was observed under the delay-distraction condition compared to the no-distraction condition. These results further validated our findings regarding the delay-distraction condition in Experiment 1 and additionally suggested that if the distractors are processed during encoding, the distraction interference can be mitigated. These findings indicate that distraction interference is not linked to the duration of exposure to the face distractors, but is primarily due to the lack of processing of the distractors at the encoding stage.

However, beyond this explanation, another potential interpretation of our results exists. Given that the face stimuli processed by participants may readily capture their attention, under the delay-distraction condition in both Experiments 1 and 2, the face distractors would immediately appear at different locations following the disappearance of the memory stimuli. This immediate appearance of face distractors might act akin to a mask, thereby disrupting the participants’ consolidation of target faces and leading to a subsequently poorer VWM performance under the delay-distraction condition. To test this possibility, we introduced a delay-gradual-distraction condition in Experiment 3. In this condition, the distractor faces do not appear immediately after the disappearance of the memory array but emerge progressively. Therefore, if the impairment in VWM performance observed in Experiments 1 and 2 under the delay-distraction condition is indeed due to the sudden appearance of face distractors during the delay stage, a reduction or elimination of distraction-induced impairment might be observed in the delay-gradual-distraction condition.

## Experiment 3

To broaden our findings and rule out the alternative hypothesis that distraction-induced interference might result from the abrupt appearance of face distractors during the delay stage, we incorporated a delay-gradual-distraction condition in Experiment 3. This condition was compared directly with the delay-distraction condition.

### Methods

#### Participants

A new sample of 26 students (six males and 20 females; mean age = 19.65 ± 1.573 years, age range: 18–24 years) participated in Experiment 3 in return for compensation. All participants reported having normal or corrected-to-normal vision, normal color vision, and no history of neurological problems. Participants provided written informed consent before participating in the study.

### Procedure

The trial structure of Experiment 3 is shown in Fig. 1. The design and procedure of Experiment 3 were identical to those of Experiment 2, except that the full-distraction condition was replaced by the delay-gradual-distraction condition. In the delay-gradual-distraction condition, only two target faces were presented in the memory array. However, following the disappearance of the memory array, two distractor faces gradually appeared over a period of 500 ms (the transparency of the distractor face images gradually decreased from 100% to 0% within this duration). Subsequently, the distractor faces remained at 0% transparency (same as the distractors in the delay-distraction condition in Experiments 1 and 2) for the next 500 ms until the test array appeared.

### Results

**Fig. 4:**
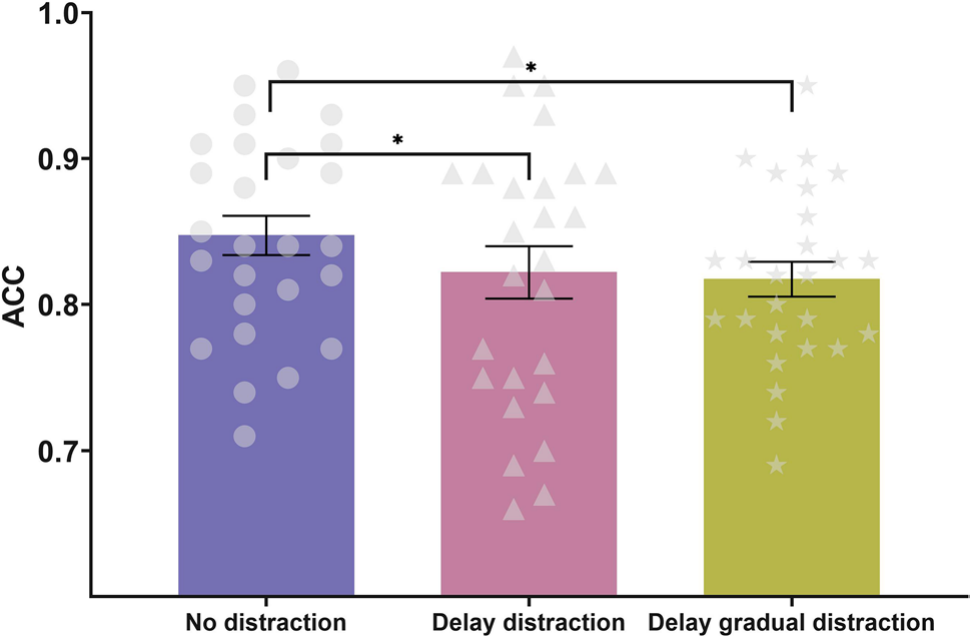
Accuracy under each condition (no-distraction condition, delay-distraction condition, and delay-gradual-distraction condition) in Experiment 3. Mean values, with error bars showing the standard error of the mean. *p < .05

The mean accuracy in each condition (no-distraction condition vs. delay-distraction condition vs. delay-gradual-distraction condition) is shown in Fig. 4. The ANOVA for the accuracy of the responses showed a significant main effect of condition, *F*(2, 50) = 4.197, *p* = 0.021, η_p_^2^ = 0.144, BF_10_ = 2.434. The accuracy was significantly higher in the no-distraction condition (*M* = 0.847, *SD* = 0.069) than in the delay-distraction condition (*M* = 0.822, *SD* = 0.091), *t*(25) = 2.395, *p*_corr_ = 0.048, Cohen’s *d* = 0.470, BF_10_ = 2.245, and in the delay-gradual-distraction condition (*M* = 0.817, *SD* = 0.061), *t*(25) = 2.668, *p*_corr_ = 0.039, Cohen’s *d* = 0.523, BF_10_ = 3.738. No significant difference was detected in the accuracy between the delay distraction and delay-gradual-distraction conditions, *t*(25) = 0.398, *p*_corr_ = 0.694, Cohen’s* d* = 0.0678, BF_10_ = 0.223.

### Discussion

The accuracy results show that the VWM performance of the participants was significantly impaired under both the delay-distraction condition and the delay-gradual-distraction condition compared to the no-distraction condition. In addition, no difference was found in the degree of impairment of VWM performance between the delay-gradual-distraction condition and the delay-distraction condition. This result thus excludes the alternative explanation that the distraction effect in the delayed-distraction condition was caused by the abrupt appearance of the face distractors.

### Exploratory correlation analysis between distraction effects and VWM capacity

Previous ERP research has demonstrated that an individual’s ability to filter face distractions is influenced by that individual’s VWM capacity, with individuals having higher VWM capacities exhibiting superior distraction filtering abilities (Ye et al., 2018). This raises an intriguing question: Is there a correlation between an individual’s VWM capacity and that individual’s susceptibility to delay distraction effects? Following each experiment in our study, the participants were asked to conduct a change detection task with color stimuli to assess their VWM capacity (as part of another study). The methodology of VWM capacity measurement is detailed in the Supplementary Materials. This enabled us to measure the VWM capacity (K) for 78 participants across the three experiments. We also computed each participant’s delay distraction effect index using the following equation:

1 $$\mathrm{Delay}\;\mathrm{distraction}\;\mathrm{effect}\;\mathrm{index}=\frac{\mathrm{ACC}\;\mathrm{in}\;\mathrm{no-} \mathrm{distraction}\;\mathrm{condition}-\mathrm{ACC}\;\mathrm{in}\;\mathrm{delay-} \mathrm{distraction}\;\mathrm{condition}}{\mathrm{ACC}\;\mathrm{in}\;\mathrm{no-} \mathrm{distraction}\;\mathrm{condition}}\times100\%.$$

A positive and larger delay distraction effect index indicates a greater degradation of VWM performance due to distraction from task-irrelevant distractors during the delay stage. A delay distraction effect index equal to zero indicates that the presence of distractors does not impact VWM performance. Conversely, a negative delay distraction effect index suggests that individuals exhibit better VWM performance under the delay distractor conditions than in the absence of distractors. If participants with higher VWM capacities demonstrate stronger resistance to distractions during the delay stage, one might anticipate a significant negative correlation between an individual’s VWM capacity and their delay distraction effect index.

### Results

**Fig. 5:**
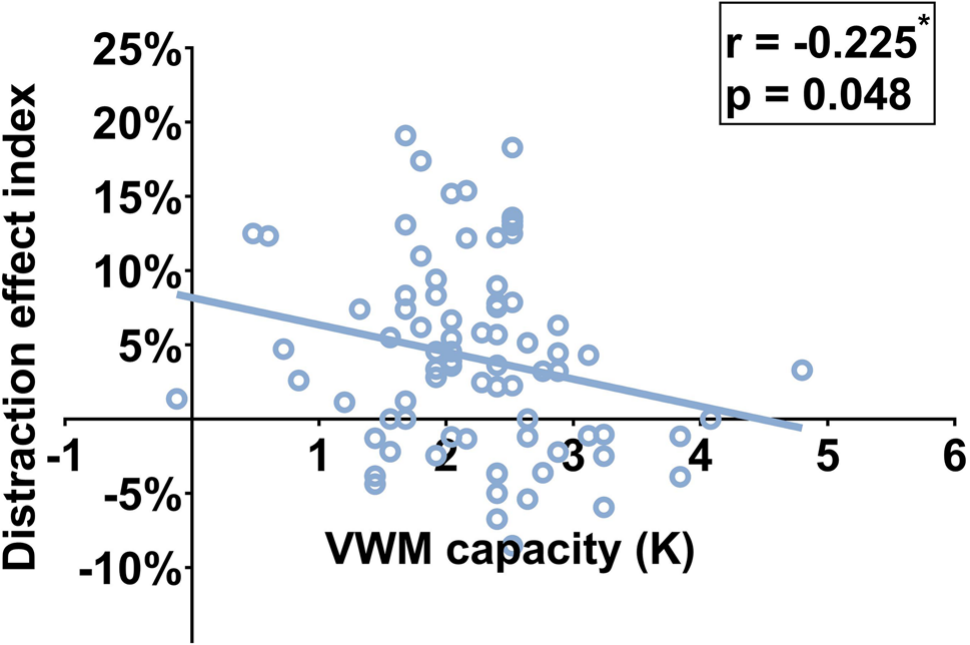
Pearson’s correlation (two-tailed) between the delay distraction effect index and VWM capacity (K). *p < .05

A correlation analysis was conducted to examine the relationship between each participant’s VWM capacity (K) and the delay distraction effect index. The results revealed a significant negative correlation between K and the delay distraction effect index (*r* = −.225, *p* = 0.048), as depicted in Fig. 5.

#### Discussion

These findings suggest that individuals with higher VWM capacities are less impaired by the presence of facial distractors during the delay stage, in agreement with our previous research finding that individuals with higher VWM capacities could filter out distractive faces more effectively (Ye et al., 2018). This result also corresponds with a previous ERP study that identified a positive correlation between an individual’s VWM capacity and the distractor positivity (PD) ERP component, reflecting the suppression process for to-be-ignored items (Feldmann-Wustefeld & Vogel, 2019).

Although the results from Experiments 1 and 2 indicated that presenting distractors during the encoding stage (the encoding-distraction condition in Experiment 1 and the full distraction condition in Experiment 2) did not impair the participants’ VWM performance, previous VWM studies using simple stimuli as memory materials found a significant correlation between VWM capacity and the filtering effect when distractors were presented alongside the target during the encoding stage (Owens et al., 2012; Vogel et al., 2005). Therefore, we conducted correlation analyses for Experiments 1 and 2 to assess the relationship between VWM capacity and the distracting effect in the encoding-distraction condition/full distraction condition. However, no significant correlation was found between individuals’ VWM capacity and the encoding-distraction effect/full-distraction effect (Experiment 1,* r* = .159, *p* = 0.437; Experiment 2, *r* = −.075, *p* = 0.717). Considering that the distractors in the full-distractor condition in Experiment 2 were also presented from the encoding stage, we combined the encoding-distraction effect from Experiment 1 and the full-distraction effect from Experiment 2 to examine their correlation with VWM capacity during the encoding stage. The results still showed no significant correlation between individual VWM capacity and the distraction effect produced during the encoding stage (*r* = .087, *p* = 0.539).

Notably, while we did not find a correlation between individuals’ VWM capacity and the distraction effect during the encoding stage, this result does not challenge the previous findings of a negative correlation between individual VWM capacity and encoding-stage-distraction effects (Owens et al., 2012; Vogel et al., 2005). We believe that the reason we did not observe a significant correlation between individual VWM capacity and encoding-distraction effects in our study is that, in the present study, the participants, regardless of their VWM capacity, were not significantly negatively impacted by distractors appearing during the encoding stage. We will further discuss the potential reasons for the absence of observed encoding-distraction effects in the present study and in the study by Duan et al. (2023) in the General Discussion.

## General discussion

The aim of this study was to assess the differential impact of face distractors during the encoding and delay stages on VWM. To this end, we presented the distractors either during the encoding stage, during the delay stage, or through both stages. In general, we found a dissociated face distraction effect between the encoding and delay conditions. Specifically, distraction interference of VWM performance was evident when the face distractors were presented exclusively during the delay stage, but not when the distractors were presented at the encoding stage. In addition, our results demonstrated that as long as the participants suppressed the face distractors during the encoding stage, the distractors would not impair the VWM performance, even if those distractors persisted until the end of the delay stage. Moreover, we proved that the impairment of VWM performance caused by face distractors during the delay stage was not due to the sudden appearance of face distractors.

Our findings are consistent with those reported by Duan et al. (2023) for simple distractors, thereby demonstrating that the resistance mechanism against simple distractors can be extended to complex real-world distractors. However, as a notable divergence from Duan et al. (2023), our investigation used distinct experimental stimuli and methods to differentiate between targets and distractors. Our results validate that even for facial information, which humans are particularly adept at processing, the distraction-related degradations only exist when face distractors are presented during the delay stage and not during encoding. This contributes to the research on distraction processing in VWM by providing results with higher ecological validity.

The occurrence of face distraction effects only during the delay stage, and not during the encoding stage, suggests that this effect may be primarily due to attention being captured by the novel face distractors presented. When both target and distractor faces are presented during the encoding stage, the participants allocate and focus their resources on the target while suppressing the distractor face information. This suppression of distractor information extends into the VWM maintenance stage (delay stage). Conversely, when distractor faces are presented only after the end of the encoding stage—by which time the encoded target stimuli have disappeared—the face distractors appearing on the screen during the delay stage become salient stimuli that now capture the participants’ attention, leading to automatic processing. The salient distractors require merely approximately 220 ms to capture an individual's attention (Lin et al., 2024), thereby impacting the maintenance of VWM representations. This ultimately results in dissociated distraction effects. To our knowledge, this study provides the first empirical evidence revealing a dissociated distraction effect of real-world stimuli on VWM performance, thereby highlighting the unique impact of introducing face distractors at different VWM stages.

A recent study by Mallett et al. (2020) also reported that facial distractors presented during the delay stage can bias VWM information, thereby supporting our findings regarding the distraction effects of the delay stage. However, Mallett et al. (2020) focused primarily on the perceptual impact of facial stimuli and did not systematically explore their differential effects at various VWM stages. Their study utilized only delay-distraction conditions without a no-distraction condition. Their setting therefore prevented a direct assessment of the damage caused by facial distractors to VWM performance. By contrast, our research, which contrasted both the delay-distraction and no-distraction conditions, offers concrete evidence for the detrimental effects of face distractors during the delay stage.

Interestingly, our previous studies (Ye et al., 2018, 2023) showed that presenting face distractors during the encoding stage could impair VWM performance; therefore, our previous findings appear contradictory to our current findings. However, the experimental designs of our previous study (Ye et al., 2018, 2023) and the current work had two noteworthy differences. First, in our previous studies (Ye et al., 2018, 2023), the distractors were presented during both the encoding array and the test array, implying that the negative impact on VWM could originate from distractions during the response stage. Second, the durations of face distractor presentation during the encoding stage were 200 ms and 500 ms in our previous studies (Ye et al., 2018, 2023), whereas we presented face distractors during the encoding stage for 1,000 ms in the current study. This longer encoding time in our current study allowed for a more thorough consolidation process in VWM.

It is important to note that the results of the present study should not be construed as evidence against the existence of distraction effects during the encoding stage, as reported in previous findings (Vogel et al., 2005; Ye et al., 2018, 2023). Previous research has shown that a 1,000-ms presentation duration allows for the top-down influence to play a key role (Sander et al., 2011), potentially accounting for the efficient filtering of distractors during the encoding stage observed in our study and in the study by Duan et al. (2023). Regarding the impact of stimulus presentation duration on VWM resource allocation, our previously proposed two-phase resource allocation model (Ye et al., 2017, 2019, 2020) posits that VWM consolidation consists of an early phase in which resources are involuntarily allocated across all stimuli to form low-precision VWM representations. This is followed by a late consolidation phase, in which resources can be voluntarily reallocated based on task requirements. Consequently, when encoding time is limited, participants might involuntarily allocate VWM resources to face distractors due to stimulus-driven processes, leading to VWM impairment. However, with sufficient encoding time, as in our current study, participants can further reallocate and focus their VWM resources on the target items through top-down control during the later consolidation phase, thereby mitigating the impact of face distractors. Thus, the duration of stimulus presentation may influence whether participants can utilize top-down control to reduce distraction effects during the encoding phase. Future research should control for the stimulus presentation duration of the memory array to gain a more comprehensive understanding of the processing mechanisms of distractions within VWM tasks.

Another explanation for why previous research identified distraction effects during the encoding stage, while our study and that of Duan et al. (2023) did not, may be the different methods used for stimulus presentation. In studies that reported encoding-distraction effects, the CDA component within ERP techniques was often used to track the quantity of VWM representations stored by the participants. This led to a scenario in the previous studies wherein both target and distractor stimuli were presented on one side of the visual hemifield (either the left or right hemifield). Conversely, in our research and that of Duan et al. (2023), the stimuli were presented bilaterally across the visual fields. Previous studies have shown superior VWM performance for stimuli presented bilaterally across visual fields than for stimuli presented in a unilateral visual field (Delvenne, 2005; Umemoto et al., 2010), a phenomenon known as the bilateral field advantage (BFA). The BFA likely arises due to the allocation of more attentional resources when items are presented in both the left and right visual fields (Zhang et al., 2018). Therefore, in our study and that of Duan et al. (2023), the bilateral presentation of the memory array could plausibly have enabled individuals to allocate more attentional resources toward enhancing target stimuli and suppressing distractor stimuli. This would result in a superior ability to filter out distractors during the encoding stage than was evident in previous research (Vogel et al., 2005; Ye et al., 2018, 2023). Future research could explore this possibility by manipulating the stimulus presentation methods.

In our previous studies (Ye et al., 2018, 2023), we also used emotional faces as distractors. Previous research suggests that emotionally salient stimuli, such as fearful faces, more readily attract attention and are more easily stored in VWM compared with neutral faces (see reviews by Gambarota & Sessa, 2019; Xu et al., 2021), possibly contributing to the observed impairment by face distractors. Furthermore, threatening face distractors are more challenging for individuals to filter out of VWM storage (Stout et al., 2013; Ye et al., 2023). Although our current study systematically investigates the distractor effect of neutral face distractors across different stages, whether individuals can avoid impairment from emotional face distractors during the encoding stage remains unclear. Future research, building on our experimental paradigm and findings, could explore the distraction effects of different types of emotional facial stimuli and their impacts under different conditions.

Recent studies have also shown that an individual’s emotional state can influence VWM processing. For instance, previous research has demonstrated that, under negative emotional states, individuals exhibit enhanced VWM precision for target stimuli (Long et al., 2020; Xie et al., 2023; Xie & Zhang, 2016), albeit with a reduced maximum capacity for VWM storage (Figueira et al., 2017). Moreover, a recent study revealed that negative emotional states can diminish an individual’s ability to suppress distractor stimuli, leading to the automatic storage of distractors in VWM (Ye et al., 2024). Therefore, future investigations could examine whether different emotional states also affect the dissociated VWM distraction effect between the encoding and delay stages.

Previous studies have also demonstrated that mental stress or mental illnesses, such as depression, anxiety, and persistent pain (Berryman et al., 2013; Maran et al., 2015; Stout et al., 2013; Xu et al., 2018, 2023; Zhou et al., 2021), can significantly impact attention and VWM. Considering how daily life is inundated with visual information and distractions, a continuous need exists for selective storing of valuable information into VWM while suppressing distracting information within it. Future research should consider ways to enhance the ecological validity of studies on distractor processing. Building on the experimental paradigm of the present study and incorporating the aforementioned factors, future research could explore individuals’ processing mechanisms for other real-world stimulus distractions and their influencing factors, thereby enriching our comprehensive understanding of distraction-processing mechanisms in daily life.

In summary, our study using human faces as complex real-world distractors indicates that the VWM performance was significantly impaired by delay-stage distractors, but remained unaffected during the encoding stage. This dissociated VWM distraction effect results from the absence of processing distractors during the encoding stage, rather than the appearance of distractors during the delay or their abrupt emergence. By demonstrating that the mechanisms of distraction resistance previously identified with simple stimuli can be extended to more complex real-world stimuli, our study contributes to a deeper understanding of the cognitive processes underpinning VWM and its resilience against distractors. Thus, our study not only advances the theoretical understanding of VWM resilience to distractions, but it also underscores the significance of considering the timing and nature of distractors in cognitive processing.

## Supplementary Information

Below is the link to the electronic supplementary material.

Supplementary file1 (DOCX 42.7 KB)

## Data Availability

The data obtained in the study are available for open access (http://doi.org/10.17605/OSF.IO/VJMD4).
