# Correction to: The differential impact of face distractors on visual working memory across encoding and delay stages

**DOI:** 10.3758/s13414-024-02912-8

**Published:** 2024-06-26

**Authors:** Chaoxiong Ye, Qianru Xu, Zhihu Pan, Qi‑Yang Nie, Qiang Liu

**Affiliations:** 1 Institute of Brain and Psychological Sciences, Sichuan Normal University, Chengdu, 610066 China; 2 Department of Psychology, University of Jyvaskyla, 40014 Jyväskylä, Finland; 3 Center for Machine Vision and Signal Analysis, University of Oulu, 90014 Oulu, Finland; 4 School of Education, Anyang Normal University, Anyang, 455000 China; 5 Centre for Cognitive and Brain Sciences, University of Macau, Macau, 999078 China

**Correction to: Attention, Perception, & Psychophysics (2024)**

10.3758/s13414-024-02895-6

In the published version of this article, there was an error in the designation of the corresponding authors. The correct corresponding authors should be Qi-Yang Nie and Qiang Liu, as indicated in the original manuscript and final proof. The published version erroneously included Chaoxiong Ye as a corresponding author. We apologize for any confusion it may have caused. The original article has been corrected.

