# Supplementary material for: The differential impact of face distractors on visual working memory across encoding and delay stages
Source: Atten Percept Psychophys. 2024 May 31;86(6):2029–41. doi: 10.3758/s13414-024-02895-6 (PMC11410854; doi:10.3758/s13414-024-02895-6)
Supplement: Supplementary file 1 — Supplementary file1 (DOCX 42.7 KB) [file 13414_2024_2895_MOESM1_ESM.docx]

**Supplementary Materials**

## Methods used for the VWM capacity measurement

After the main task, the participants need to conduct a behavioral measurement with a color change detection task to assess their individual VWM capacity.

#### Materials

In each memory array, six squares with different colors were randomly selected from a color pool of seven colors (red: 255, 0,0; green: 0, 210, 0; blue: 0, 0, 210; orange: 228, 108, 10; yellow: 210, 210, 0; violet: 112, 48, 160; pink: 255, 75, 186) and were presented within an invisible 9.8°×7.3° rectangle centered on the fixation, against a gray background (6.1 cd/m^2^, RGB: 128, 128, 128) on a 21-inch LCD monitor (refresh rate 75 Hz) at a viewing distance of 60 cm. A single square subtended a visual angle of about 0.65° and the two squares sustained an interval of at least 1.5°. The capacity task was programmed via E-prime 1.0.

#### Procedure

At the beginning of the capacity task, the memory array consisted of six colors and was displayed for 200 ms, followed by an interval with only a fixation for 900 ms. The test array began next, and one colored square appeared at one of the six locations occupied by colors in the memory array (as in Figure S1). The participants were asked to identify whether the color in the test array was identical to the color at the same location in memory, and to press “j” if the color had changed; otherwise, they should press “f.” The color in the test array in half the trials was identical to that of the memoranda, whereas the other half changed. The test array would not disappear unless the participant pressed the keyboard or 2500 ms passed. This capacity task contained 100 trials and the total duration was about ten minutes.

**Figure S1.** An example of the procedure of VWM capacity measurement with changed test stimuli.

#### Data analyses

The VWM capacity (K) of each participant was quantified based on their results in the VWM capacity measurement. The standard formula proposed by [Cowan (2001](#_ENREF_1)) was applied: K = N × (H − F), where K is the VWM capacity, N is the size of the array (i.e., six in the present study), H is the hit rate (i.e., the proportion of correct responses when a change is present), and F is the false alarm rate (i.e., the proportion of incorrect responses when no change is present).

## Reference

Cowan, N. (2001). The magical number 4 in short-term memory: A reconsideration of mental storage capacity. *Behav Brain Sci*, *24*(1), 87-185. https://doi.org/10.1017/S0140525X01003922
